# PPL2ab neurons restore sexual responses in aged *Drosophila* males through dopamine

**DOI:** 10.1038/ncomms8490

**Published:** 2015-06-30

**Authors:** Shu-Yun Kuo, Chia-Lin Wu, Min-Yen Hsieh, Chen-Ta Lin, Rong-Kun Wen, Lien-Cheng Chen, Yu-Hui Chen, Yhu-Wei Yu, Horng-Dar Wang, Yi-Ju Su, Chun-Ju Lin, Cian-Yi Yang, Hsien-Yu Guan, Pei-Yu Wang, Tsuo-Hung Lan, Tsai-Feng Fu

**Affiliations:** 1 Department of Applied Chemistry, National Chi Nan University, 54561 Nantou, Taiwan; 2 Department of Biochemistry and Graduate Institute of Biomedical Sciences, College of Medicine, Chang Gung University, 33302 Taoyuan, Taiwan; 3 Department of Medical Research, Chang Gung Memorial Hospital, 33305 Taoyuan, Taiwan; 4 Department of Medical Laboratory Science and Biotechnology, Chung Hwa University of Medical Technology, 70703 Tainan, Taiwan; 5 Institute of Biotechnology, Institute of Systems Neuroscience, and Department of Life Science, National Tsing Hua University, 30013 Hsinchu, Taiwan; 6 Graduate Institute of Brain and Mind Sciences, College of Medicine, National Taiwan University, 10051 Taipei, Taiwan; 7 Department of Psychiatry, School of Medicine, National Yang Ming University, 11221 Taipei, Taiwan; 8 Department of Psychiatry, Taichung Veterans General Hospital, 40705 Taichung, Taiwan

## Abstract

Male sexual desire typically declines with ageing. However, our understanding of the neurobiological basis for this phenomenon is limited by our knowledge of the brain circuitry and neuronal pathways controlling male sexual desire. A number of studies across species suggest that dopamine (DA) affects sexual desire. Here we use genetic tools and behavioural assays to identify a novel subset of DA neurons that regulate age-associated male courtship activity in *Drosophila*. We find that increasing DA levels in a subset of cells in the PPL2ab neuronal cluster is necessary and sufficient for increased sustained courtship in both young and aged male flies. Our results indicate that preventing the age-related decline in DA levels in PPL2ab neurons alleviates diminished courtship behaviours in male *Drosophila*. These results may provide the foundation for deciphering the circuitry involved in sexual motivation in the male *Drosophila* brain.

## Abstract (web summary)

We currently lack a detailed understanding of the neurobiological basis for the decline of male sexual desire with age. Here the authors demonstrate that restoring impaired dopaminergic signalling in a specific cluster of neurons in the *Drosophila* brain increases sexual behaviour in ageing male flies.

## Graphical abstract

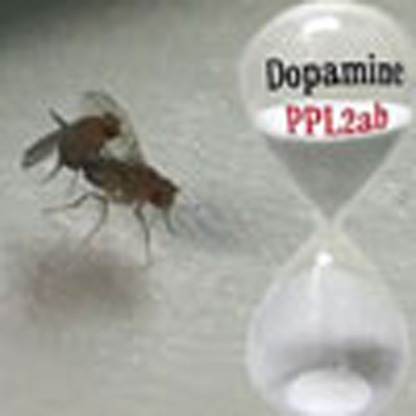

Dopamine (DA) play an important role in motor control^1^, motivation^2^,^3^, circadian^4^, cognition^2^,^5^ and reward^3^. The regulation of sexual gratification by DA in mammals has also been well described^6^,^7^,^8^. Moreover, evidence that DA mediates regulates human sexual behaviours comes from cases of inadvertent hypersexuality resulting from DA treatment in patients with Parkinson's disease^9^. Despite these advances in our understanding of the role of DA in controlling sexual desire in mammals, the neurobiological basis of this phenomenon at the cellular and circuit level is limited.

Sexual function typically declines in old age^10^,^11^,^12^. Ageing is characterized by physiological, pathological, behavioural, and psychosocial changes. All of these factors affect sexual function, and it is difficult to disentangle their individual effects. The cellular and molecular mechanisms underlying sexual decline with age have been difficult to study and remain poorly understood. Advances in genetic and behavioural tools available for studying *Drosophila*, coupled with a more detailed understanding of *Drosophila* courtship, have now afforded powerful methods to study essential functions and regulatory mechanisms of sexuality in these animals.

Previous studies using *Drosophila* demonstrate that various physiological functions including courtship behaviour are affected by ageing^13^,^14^,^15^. In the *Drosophila* brain, there are ∼280 DA neurons whose cell bodies are organized into at least 13 clusters^16^. We hypothesized that these DA neurons would contain subpopulations that regulate sexuality and would thus provide a powerful model to understand how male sexual responses are regulated by DA pathways in the brain over the lifespan. Here we demonstrate that DA levels in the protocerebral posteriolateral dopaminergic cluster neuron 2ab (PPL2ab) regulate male courtship sustainment and that tyrosine hydroxylase (TH), an enzyme responsible for DA synthesis, levels in these cells decline with age. Interestingly, altering DA levels in specific PPL2ab neurons did not affect motor activity, sensory processing (including smell and taste), nor the length of life, suggesting that PPL2ab neurons specifically regulate male sexuality. Together, our results suggest a neurobiological mechanism for the decline of sexuality in ageing and advance our understanding of how the brain regulates male libido levels and sexual motivation.

## Results

### PPL2ab neurons regulate sexuality in male flies

**Figure 1:**
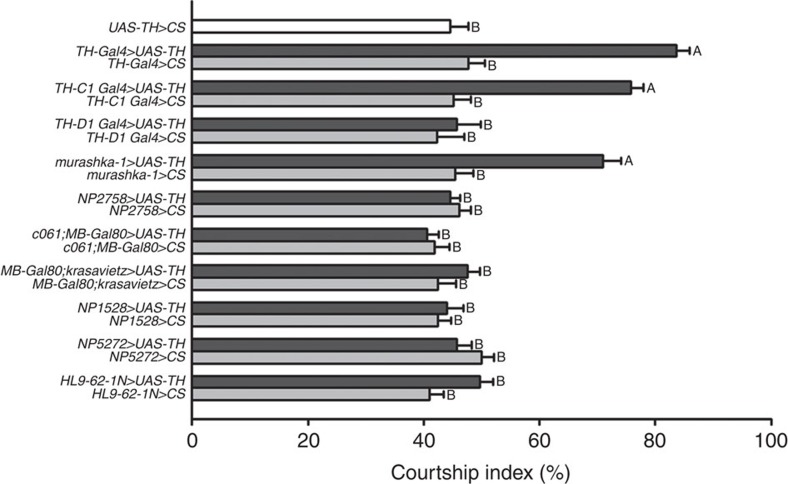
A small subset of DAergic neurons regulates male courtship activity. TH expression was driven by *Gal4* drivers with restricted expression in specific DA neurons and the male courtship index was obtained in flies from each line (see below for details). The results are presented in the bar plot. ANOVAs were used to compare genetically manipulated 10-day-old male flies across the different genetic manipulations or with the relevant heterozygous controls. Significant differences were observed in the courtship index in *TH-Gal4>UAS-TH*, *TH-C1-Gal4>UAS-TH*, and *murashka-1-Gal4>UAS-TH* flies compared with the corresponding *Gal4* driver and *UAS-TH* heterozygous flies. Each column represents the mean of 22 tests. The error bars indicate+s.e.m. The means within each column followed by the same letters are not significantly different at the threshold of *P*<0.05 by a one-way ANOVA followed by Tukey's test. See Supplementary Figs 1 and 2, and Supplementary Table 1 for representative images and a summary of the driver expression patterns. Genotypes: (1) *+/y;+/+;+/UAS-TH*; (2) *+/y;+/+;TH-Gal4/UAS-TH*; (3) *+/y;+/+;TH-Gal4/+*; (4) *+/y; TH-C1-Gal4/+; +/UAS-TH*; (5) *+/y; TH-C1-Gal4/+; +/+*; (6) *+/y; TH-D1-Gal4/+; +/UAS-TH*; (7) *+/y; TH-D1-Gal4/+; +/+*; (8) *+/y;+/+;murashka-1-Gal4/UAS-TH*; (9) *+/y;+/+;murashka-1-Gal4/+*; (10) *NP2758-Gal4/y;+/+;+/UAS-TH*; (11) *NP2758-Gal4/y;+/+;+/+*; (12) *c061-Gal4/y;MB-Gal80/+;+/UAS-TH*; (13) *c061-Gal4/y;MB-Gal80/+;+/+*; (14) *+/y;MB-Gal80/+;krasavietz-Gal4/UAS-TH*; (15) *+/y;MB-Gal80/+;krasavietz-Gal4/+*; (16) *+/y;NP1528-Gal4/+;+/UAS-TH*; (17) *+/y;NP1528-Gal4/+;+/+*; (18) *+/y;NP5272-Gal4/+;+/UAS-TH*; (19) *+/y;NP5272-Gal4/+;+/+*; (20) *+/y;+/+;HL9-62-1N-Gal4/UAS-TH*; and (21) *+/y;+/+;HL9-62-1N-Gal4/+*.

**Figure 2:**
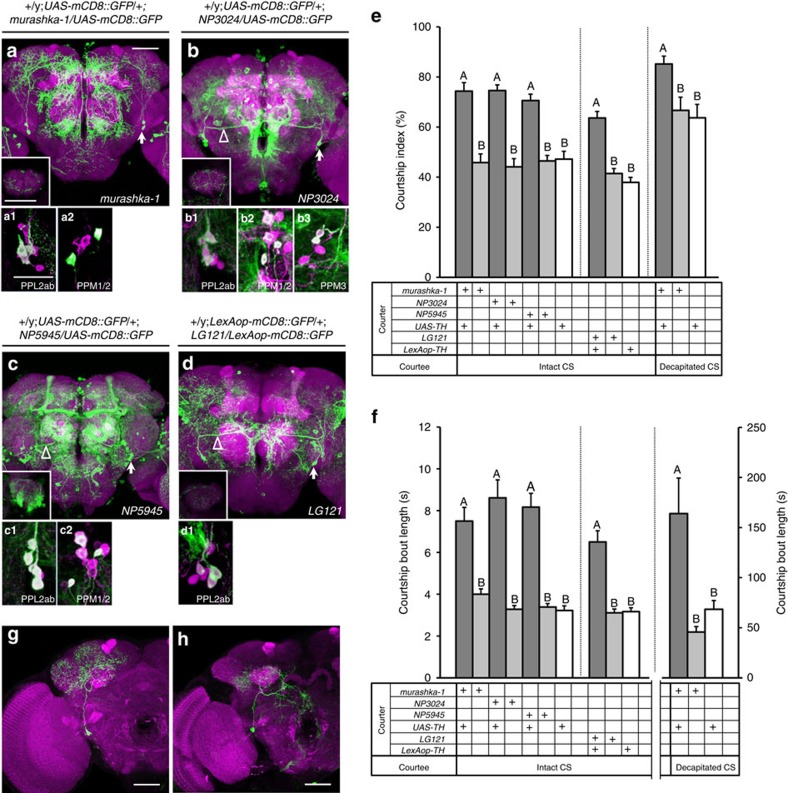
DAergic PPL2ab neurons regulate male courtship sustainment. Representative images showing the expression pattern of the *Gal4* driver lines: (**a**) *murashka-1-Gal4*, (**b**) *NP3024-Gal4*, (**c**) *NP5945-Gal4* and (**d**) *LG121-LexA*; driver expression patterns revealed by *UAS-mCD8::GFP* (or *LexAop-mCD8::GFP*) in the adult brain (10-day-old) are shown (green in **a**–**d** shows the PPL2ab cell bodies indicated by the arrows and POT track indicated by the arrowheads; the inset box shows the expression pattern in a single calyx). The neuropil was immunostained with an anti-DLG antibody (magenta in **a**–**d**). The cell bodies of PPL2ab neurons (green in a1-d1) were labelled with four independent drivers; PPM1/2 neurons (green in a2-c2) were labelled by *murashka-1-Gal4*, *NP3024-Gal4* and *NP5945-Gal4*; and PPM3 neurons (green in b3) were labelled by the *NP3024-Gal4*; all of these cell were DAergic as indicated by TH-antibody immunostaining (magenta in a1-2, b1-3, c1-2 and d1). Scale bars, 20 μm. (**e**,**f**) The results for courtship index and courtship bout length towards 3-day-old intact CS females or decapitated CS females are shown. ANOVAs were used to compare genetically manipulated 10-day-old male flies (*murashka-1-Gal4>UAS-TH*, *NP3024-Gal4>UAS-TH*, *NP5945-Gal4>UAS-TH*, and *LG121-LexA>LexAop-TH*) with the corresponding driver and effector heterozygous controls. Significant differences in courtship index (**e**) and courtship bout length (**f**) were observed. Each column represents the mean of 18 tests. The error bars indicate+s.e.m. The means for columns followed by the same letters were not significantly different according to the *P*<0.05 threshold by a one-way ANOVA followed by Tukey's test per grouped columns (separated by a dashed line). (**g**,**h**) Two types of PPL2ab neurons from *murashka-1-Gal4* flip-out clones (green) and neuropil were immunostained with the anti-DLG antibody (magenta). Genotypes in (**a**–**f**): (1) *+/y;+/+;murashka-1-Gal4/UAS-TH*; (2) *+/y;+/+;murashka-1-Gal4/+*; (3) *+/y;+/+;NP3024-Gal4/UAS-TH*; (4) *+/y;+/+;NP3024-Gal4/+*; (5) *+/y;+/+;NP5945-Gal4/UAS-TH*; (6) *+/y;+/+;NP5945-Gal4/+*; (7) *+/y;+/+;+/UAS-TH*; (8) *+/y;LexAop-TH/+;murashka-1-Gal4/+*; and (9) *+/y;LexAop-TH/+;+/+.* Genotypes in (**g**,**h**): *hs-FLP/y; +/+; UAS>rCD2,y^+^>mCD8::GFP/murashka-1-Gal4*.

To determine the specific DA neurons that regulate *Drosophila* courtship sustainment, we used Gal4 drivers to manipulate DA activity in restricted groups of DAergic neurons in the fly brain (Supplementary Fig. 1; *murashka-1-Gal4* driver in Fig. 2a; and summarized in Supplementary Table 1). We then analysed the effect of cell-type-specific DA overexpression on courtship intensity in male flies (Fig. 1). Our results revealed that increased expression of TH in *TH-Gal4-*(ref. 17) and *TH-C1-Gal4-*positive^18^ neurons significantly enhanced the male courtship index (Fig. 1). Interestingly, we observed similar results by overexpressing TH in *murashka-1-Gal4*-positive neurons, which targets a more restricted cell population (Fig. 1). To label the cells targeted by *TH-Gal4*, *TH-C1-Gal4* and *murashka-1-Gal4,* we crossed each line to the *UAS-mCD8::GFP* reporter line and counterstained each by TH immunohistochemistry. By assessing the anatomical profile of TH cells coexpressing GFP in each line, we found that expression overlapped between the three Gal4 drivers in a subset of DA neurons in the PPL2ab (Fig. 2a1 and Supplementary Fig. 1g1) and the protocerebral posteriomedial dopaminergic cluster neuron 1/2 (PPM1/2) (Fig. 2a2 and Supplementary Fig. 1g3). These data suggested that male courtship intensity might be regulated by DA release from these neurons.

To clarify whether PPL2ab or PPM1/2 neurons are involved in male courtship behaviour, we examined the effect of selectively overexpressing TH and GFP from two Gal4 drivers and one LexA driver expressed in these regions. We found that *NP3024-Gal4* and *NP5945-Gal4* were partially expressed in the PPL2ab and PPM1/2 clusters (Fig. 2b,c), while *LG121-LexA* was expressed only in the PPL2ab cluster (Fig. 2d). We observed that the cell bodies of PPL2ab neurons labelled by these drivers were located on the lateral edge of the protocerebrum near the optical lobes (indicated by arrows in Fig. 2a–d), and projected ascending neural fibres to the calyx (CX) and the lateral horn (LH). TH-positive PPL2ab neurons were labelled by four independent drivers used in this study (*murashka-1-Gal4*, *NP5945-Gal4*, *NP3024-Gal4* and *LG121-LexA*) (Figs 2a1–d1), and importantly the expression patterns of these four drivers overlapped only in PPL2ab neurons. To determine whether these PPL2ab neurons regulate courtship behaviour, we overexpressed TH using each driver and carried out behavioural assays. We observed a significant increase in the courtship index and courtship bout length towards either intact or decapitated (that is, immobilized females) target females in all lines (one-way ANOVA followed by Tukey's test, *P*<0.05) (Fig. 2e,f). The use of decapitated target females provides a method to evaluate whether male sexual attraction and enhanced courtship sustainment are regulated by increased DA levels in PPL2ab neurons. By using an immobilized female, we removed the reciprocal effects of male–female interactions. Our results therefore demonstrate that PPL2ab neurons specifically affected male courtship intensity through DA, rather than their attractiveness.

There are approximately six neurons in the PPL2ab cluster in each brain hemisphere, which in previous studies^13^ have been classified into at least four subtypes based on their fibre innervations. Two subtypes of PPL2ab neurons project to the ipsilateral CX and LH; one innervates the entire LH, while the other innervates only the ventral portion. Another subtype innervates the lobula and extends a dorso-medial branch that travels along the posterior optical track (POT) and ramifies near the dorsal end of oesophagus. The remaining subtype innervates the posterior lateral protocerebrum^13^. Among the PPL2ab driver lines we used, three (*NP3024-Gal4*, *NP5945-Gal4* and *LG121-LexA*) exhibited distinct POT track expression patterns (indicated by arrowheads in Fig. 2b–d), while *murashka-1-Gal4* was not expressed in the POT. This implied that PPL2ab innervations of the POT might be dispensable for regulating male courtship intensity.

To confirm which other subtypes of PPL2ab neurons are likely necessary for male courtship sustainment, the *murashka-1-Gal4* line was used to drive the *UAS*>*CD2,y^+^>CD8::GFP* transgene combined with an *Hs-flp* transgene. Two different subtypes of PPL2ab neurons in *murashka-1-Gal4* were deciphered in flip-out clones (Fig. 2g,h). One of the clones contained cells with ascending branches of neurites innervating the CX, and a second branch of neurites that innervated the entire LH and superior medial protocerebrum (Fig. 2g; Supplementary Movie 1). Another clone of cells projected one branch of primary neurites to the CX, and another branch to the adjacent posterior inferior lateral protocerebrum and the ventral portion of the LH; a third branch projected medially and innervated the middle inferior medial protocerebrum (Fig. 2h; Supplementary Movie 2). When considered in conjunction with our behavioural results, these anatomical expression data indicated that the elevated DA levels elicited in a subset of *murashka-1-Gal4* expressing PPL2ab neurons in these regions was associated with courtship sustainment in young adult flies (10 days old).

### DA levels affect courtship sustainment in aged flies

Senescence is a phenomenon that involves a decline in physiological functions over time because of the deleterious side effects of the continuous metabolic reactions in a living body. It has been reported that ageing influences sexuality and sexual desire^10^. To determine whether courtship sustainment changes as *Drosophila* age, we analysed courtship behaviours in wild-type Canton S (CS) males at different ages (5–60 days of age) and found that the maximum courtship index and courtship bout length occurred at the age of 10 days in adult males (Supplementary Fig. 2a,b). The results also revealed that courtship sustainment gradually decreased over ageing from the maximum at 10 days old. Given that motor activity and courtship activity are intimately related, we next examined motor activity associated with age in flies. Negative gravitactic behaviour analysis (a climbing assay)^19^ demonstrated that climbing activity persists across most of the lifespan before declining in 50-day-old male flies (Supplementary Fig. 2c). We utilized the *Drosophila* activity monitoring (DAM) system^20^ to record spontaneous motor activities of 10- and 40-day-old males for 24 h and demonstrated that there were no significant differences observed between the two groups (Supplementary Fig. 2d). However, a more than 10-fold reduction of DA level in tissue samples of the heads of 40-day-old aged males was observed, as compared with 10-day-old mature males (Supplementary Fig. 3a,b). We then confirmed by TH immunostainings that TH protein levels were reduced in PPL2ab neurons in aged males (Supplementary Fig. 3c). In addition, the numbers of PPL2ab neurons was the same in aged flies indicating that these neurons did not degenerate during ageing (Supplementary Fig. 3d). We thus demonstrated that DA levels in the fly brain gradually decreased with increasing age, including in PPL2ab neurons. Notably, at least in the PPL2ab clusters, this was not the result of age-related neuronal degeneration in the 40-day-old aged fly brain.

**Figure 3:**
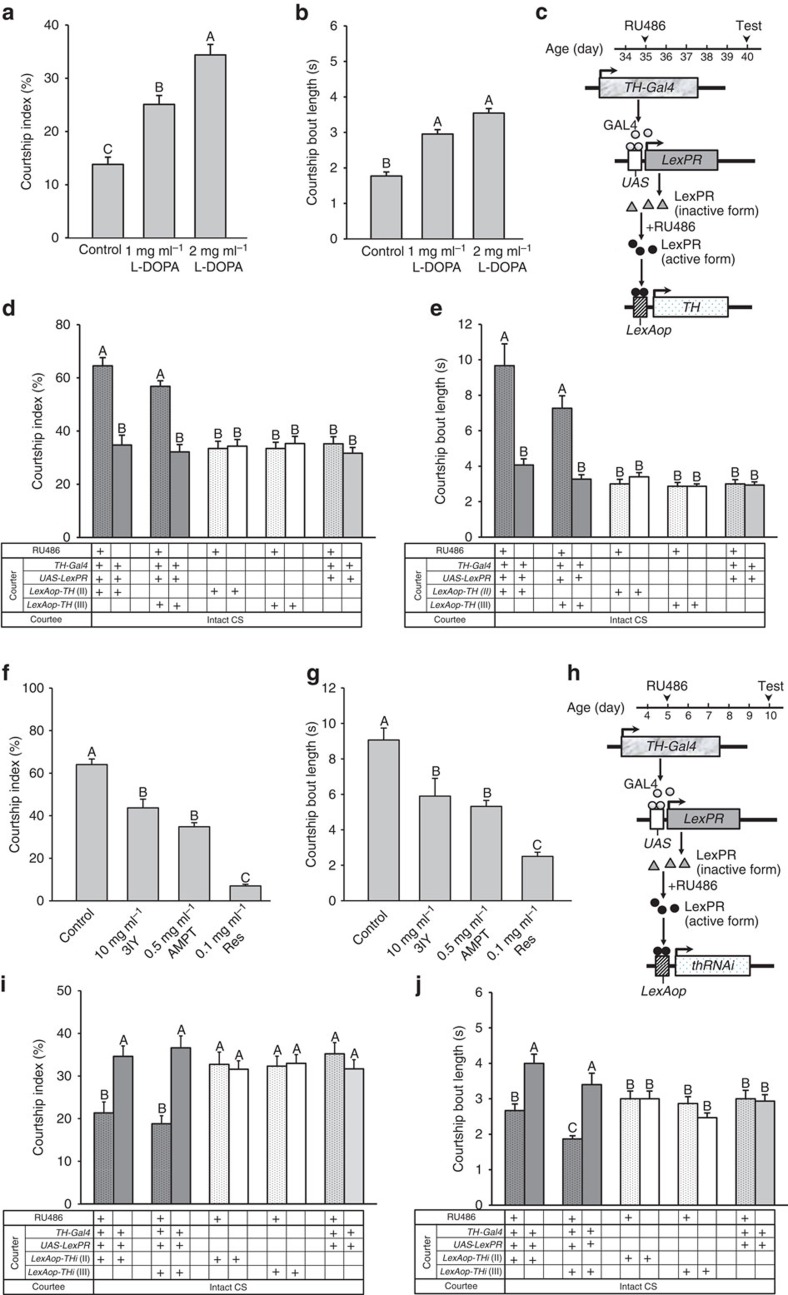
DA levels affect male courtship sustainment in aged flies. CS males were fed L-Dopa for 5 days beginning 35 days post-eclosion to increase DA levels. Behavioural assessments were carried out at 40 days post-eclosion. Significant differences were observed in the courtship index (**a**) and courtship bout length (**b**) in the flies fed 1 or 2 mg ml^−1^ L-DOPA compared with the L-DOPA-naive controls. Each column represents the mean of 22 tests. (**c**–**e**) Schematic of the LexPR/*LexAop* inducible system; it was used to temporally increase DA levels in *TH-GAL4*-expressing neurons. Males aged 35 days were fed 1.5 mM RU486 (+) for 5 days and tested for the courtship behaviours at the 40-day-old time point (**c**). Significant differences were found in the courtship index (**d**) and courtship bout length (**e**) in the flies that carried the *+/y;UAS-LexPR/LexAop-TH;+/TH-Gal4* or *+/y;UAS-LexPR/+;TH-Gal4/LexAop-TH* transgenes as compared with the flies of the same genetic background that did not receive RU486 treatment. Each bar represents the mean of 15 tests. (**f**–**j**) CS males at 5 days post-eclosion were fed 3IY, AMPT or Res for 5 days. Courtship behaviour analysis was conducted at the 10-day-old time point. There were significant differences in the courtship index (**f**) and courtship bout length (**g**) in the treated flies compared with non-treated controls. Each column represents the mean of 22 tests. (**h**–**j**) The *LexPR/LexAop* inducible system is diagrammed; it was used to temporally decrease DA levels in *TH-Gal4* expressing neurons. Male flies aged 5 days were fed 1.5 mM RU486 (+) for 5 days and tested for the courtship behaviours at the 10-day-old time point (**h**). There were significant differences in the courtship index (**i**) and courtship bout length (**j**) in the flies that carried the *+/y;UAS-LexPR/+;TH-Gal4/LexAop-thRNAi* or *+/y;UAS-LexPR/LexAop-thRNAi;+/TH-Gal4* transgenes compared with flies of the same genotype without RU486 treatment. Each column represents the mean of 15 tests. The error bars indicate+s.e.m. The means within a column followed by the same letters are not significantly different at the threshold of *P*<0.05 by two-way ANOVA followed by a Bonferroni multiple-comparisons test.

To examine the effect of increased DA levels in aged flies on courtship sustainment, we fed 35-day-old male flies with L-3,4-dihydroxyphenylalanine (L-DOPA; the precursor for DA synthesis) for 5 days and tested courtship sustainment on day 40. Interestingly, courtship sustainment in flies that were fed L-DOPA was significantly upregulated in a dose-dependent manner, as compared with the control (two-way ANOVA followed by a Bonferroni multiple-comparisons test, *P*<0.05). Correspondingly, the courtship index and courtship bout length were significantly increased in files that were fed L-DOPA (two-way analysis of variance (ANOVA) followed by a Bonferroni multiple-comparisons test, *P*<0.05) (Fig. 3a,b). To carry out a neuron-specific assay of the effects of increased DA expression, we induced extra TH production, and hence more DA synthesis, within DAergic neurons in 35-day-old males by feeding drug-sensitive mutant flies engineered with the *LexPR*/*LexAop* gene expression technique^21^ (*UAS-LexPR;TH-Gal4*>*LexAop-TH*) (Fig. 3c) 1.5 mM RU486 for 5 days. We found that the courtship index and courtship bout length of 40-day-old males were significantly increased in drug-treated flies (Fig. 3d,e). In contrast, when we inhibited DA function by feeding mature flies with α-methyl-*p*-tyrosine (AMPT) or 3-iodo-tyrosine (3IY) (both potent inhibitors of TH) and reserpine (Res; an inhibitor of vesicular monoamine transporter) for 5 days, we observed a marked reduction in the courtship index and courtship bout length (two-way ANOVA followed by a Bonferroni multiple-comparisons test, *P*<0.05) (Fig. 3f,g ). In flies harbouring the same gene switch system, we disrupted DA production in young flies by RNAi-mediated knockdown of *th* transgene in DAergic neurons (*UAS-LexPR;TH-Gal4*>*LexAop-thRNAi*) (Fig. 3h), and this phenocopied the effects of DA inhibitor administration (two-way ANOVA followed by a Bonferroni multiple-comparisons test, *P*<0.05) (Fig. 3i,j). Together, these data indicate that DA release from *TH-Gal4*-positive neurons is necessary and sufficient to promote strong male courtship behaviour.

### PPL2ab neurons modulate male sexuality in aged flies

**Figure 4:**
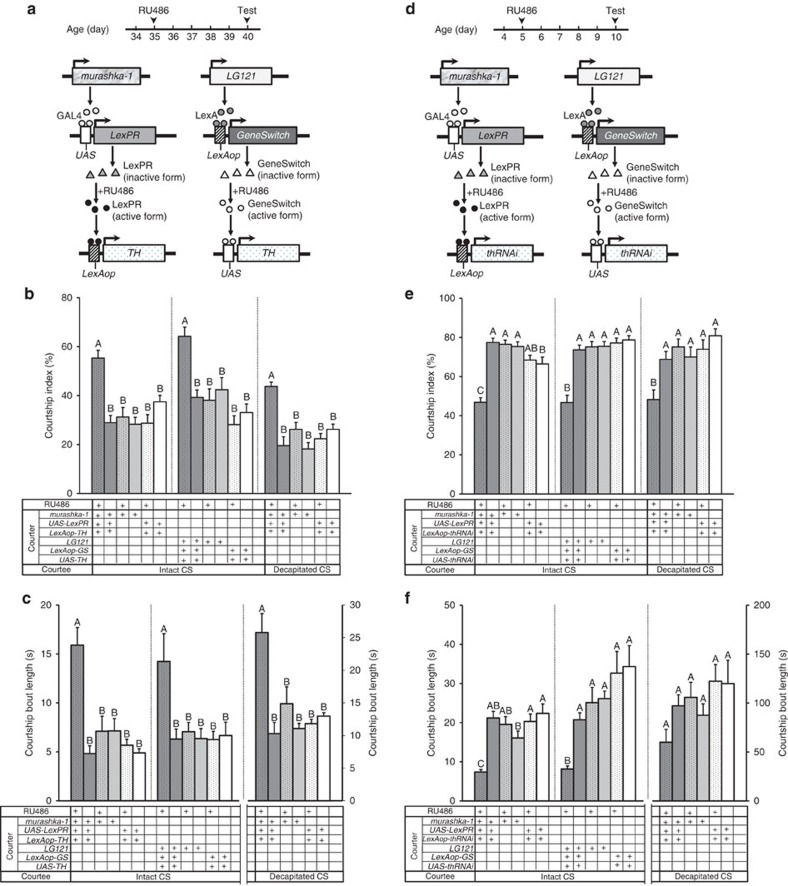
PPL2ab neurons affect male courtship sustainment through DA. (**a**) A diagram of the LexPR/*LexAop* (or GeneSwitch/*UAS*)-inducible system, which was used to temporally increase DA levels specifically in Gal4- and LexA-expressing neurons. Males aged 35 days were fed 1.5 mM RU486 (+) for 5 days, and the courtship strength was tested at the 40-day-old time point. There were significant differences in the courtship index (**b**) and courtship bout length (**c**) in the flies that received RU486 and carried the *murashka-1-Gal4>UAS-LexPR; LexAop-TH* or *LG121-LexA>LexAop-GeneSwitch;UAS-TH* compared with flies of the same genotype that did not receive RU486 treatment, as well as for the corresponding heterozygous driver and effector genotypes. Each column represents the mean of 15 tests. (**d**) Decreased DA levels brought about in the *LexPR*/*LexAop-thRNAi* (or *GeneSwitch*/*UAS-thRNAi*)-inducible system in the PPL2ab neurons inhibited mature male courtship sustainment. Males aged 5 days were fed 1.5 mM RU486 (+) for 5 days and tested for courtship strength at 10 days old. We did not observe any significant differences in the courtship index (**e**) and courtship bout length (**f**) in the flies that carried *murashka-1-Gal4; UAS-Dcr2>UAS-LexPR;LexAop-thRNAi* or *LG121-LexA;UAS-Dcr2>LexAop-GeneSwitch;UAS-thRNAi* as compared with flies of the same genotype that did not receive RU486 treatment, as well as for the corresponding heterozygous driver and effector genotypes. Each column represents the mean of 15 tests. The error bars indicate+s.e.m. The means for columns followed by the same letters were not significantly different at the threshold of *P*<0.05 by two-way ANOVA followed by a Bonferroni multiple-comparisons test per grouped columns (separated by a dashed line).

We wondered whether the diminished courtship sustainment in aged male flies might be restored by increasing the DA levels in PPL2ab neurons. We temporally induced DA expression in PPL2ab neurons from day 35 to 40 post-eclosion and tested courtship sustainment in the 40-day-old flies (Fig. 4a), we found that the courtship index and courtship bout length were significantly increased in aged males (two-way ANOVA followed by a Bonferroni multiple-comparisons test, *P*<0.05) (Fig. 4b,c). In contrast, when we induced downregulated DA levels in PPL2ab neurons from day 5 to 10 post-eclosion and tested courtship sustainment in 10-day-old flies (Fig. 4d), we observed a significant decrease in both the courtship index and courtship bout length in mature males (two-way ANOVA followed by a Bonferroni multiple-comparisons test, *P*<0.05) (Fig. 4e,f). There was a non-significant trend for decreasing courtship bout length towards the decapitated target female in flies with downregulated *th* in LG121 neurons versus the control group (two-way ANOVA followed by a Bonferroni multiple-comparisons test, *P*>0.05).

To further confirm that the above results could be fully explained by DA signalling, we conducted several control and neuronal activity assays. The effectiveness of all TH effectors used in this study was confirmed by RT-qPCR and TH-immunostaining methods, and in each case, we observed a significantly altered amount of *th* transcript and TH protein in flies (Supplementary Fig. 4). In addition, we used ReaChR (red-activatable ChR)^22^,^23^ to modulate the activity of PPL2ab neurons by fluorescent red light luminescence during fly courtship assays. Courtship sustainment was significantly increased in experimental groups with light activated PPL2ab neurons compared with the controls, in accordance with our results obtained by directly increasing DA levels (one-way ANOVA followed by Tukey's test, *P*<0.05) (Supplementary Fig. 5). Furthermore, we observed a significantly increased courtship index and courtship bout length in both intact females and decapitated female targets.

### Two pairs of PPL2ab neurons affect sexuality in aged males

**Figure 5:**
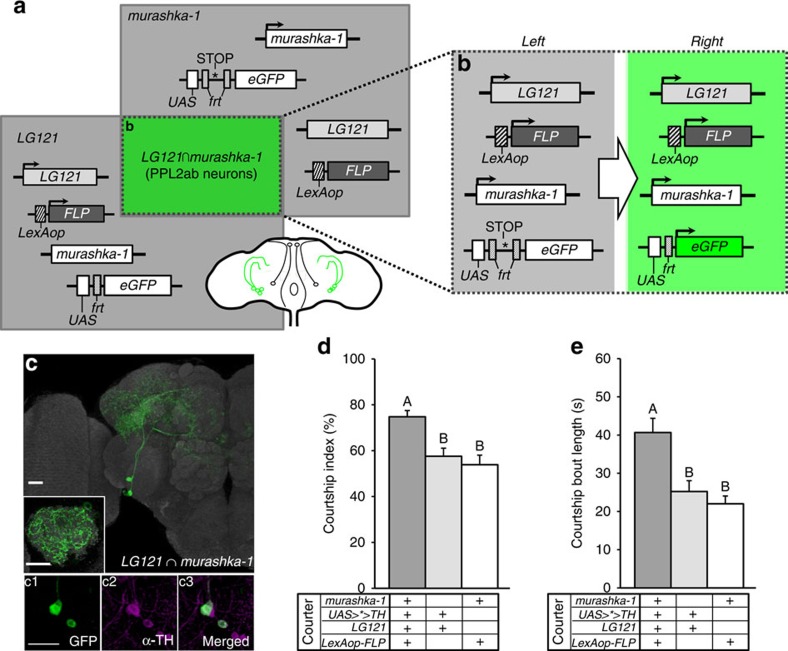
An intersectional genetic approach identified two subtypes of TH-positive PPL2ab neurons that enhance courtship sustainment. (**a**) Diagrams explaining the genetic targeting strategies to restrict the driver expression specifically to PPL2ab neurons using an intersectional LexA-induced FLP recombinase (FLP) and Gal4 intersectional methods. The two grey boxes represent the extent of the *murashka-1-Gal4* or *LG121-LexA* expression pattern. The drivers/cell types targeted are illustrated in the overlapping boxes from left to right. The resulting overlapping cell populations that express GFP are indicated in the intersecting green square. (**b**) *eGFP* expression is limited to the regions where both FLP and Gal4 are expressed. The scenario indicated by the green shaded box indicates that GFP is expressed when LexA induces *LexAop-FLP* expression, as the transcriptional stop cassette is removed allowing for GAL4-induced expression (the *Right* green square). (**c**) A representative image showing the GFP expression pattern resulting from the intersectional *murashka-1-Gal4* and *LG121-LexA* drivers. Note, the *UAS>eGFP* reporter is only expressed in the subset of PPL2ab neurons that co-expressed both GAL4 and LexA (a single calyx is magnified in the inset). (c1-3) Demonstration that the cell bodies of PPL2ab neurons in *LG121-LexA*∩*murashka-1-Gal4* (green in c1) are dopaminergic, as indicated by TH-antibody immunostaining (magenta in c2; merged in c3). Scale bar, 20 μm. (**d**,**e**) The genetic intersectional approach was applied to restrict TH overexpression in these two pairs of PPL2ab neurons. Courtship sustainment of 40-day-old males towards 3-day-old intact CS females was analysed by determining the courtship index (**d**) and courtship bout length (**e**). Significant differences in the courtship index of the *LexAop-FLP;+; murashka-1-Gal4* X UAS>*>TH; LG121-LexA flies compared with the corresponding parental heterozygous flies were observed. Each column represents the mean of 17 tests. The error bars indicate+s.e.m. The means for each column followed by the same letters were not significantly different at the threshold of *P*<0.05 by a one-way ANOVA followed by Tukey's test per grouped columns (separated by a dashed line).

Given that the expression patterns of the four PPL2ab drivers we used in this study are quite broad and considering previous findings implying that PPL2ab neurons function in the regulation of male sexual sustainment, the evidence provided by the experiments to this point was merely correlative. Therefore, we used an intersectional genetic strategy to precisely manipulate DA levels of PPL2ab neurons to establish cause-and-effect relationships between the DA levels of PPL2ab neurons and courtship behaviour in male flies. Specifically, we applied a LexA-induced FLP*/frt* recombination method that was optimized to induce targeted TH misexpression in an intersectional region defined by overlapping GAL4 and FLPase activities (Fig. 5a). This strategy works as follows: FLPase driven by LexA removes either the *frt-stop-frt* (>*>) from *UAS-frt-stop-frt-mCD8::GFP* (UAS>*> GFP) or the *frt-stop-frt* (>*>) from *UAS-frt-stop-frt-TH* (UAS>*>TH) in *LG121* expressing neurons (Fig. 5b). Then, *UAS-mCD8::GFP* is activated by GAL4 only in PPL2ab neurons that are located in the regions targeted by both *LG121-LexA* and *murashka-1-Gal4* (*LG121*∩*murashka-1*) (Fig. 5c). We then used these flies for further behavioural studies. After applying intersectional targeting to specific PPL2ab neurons, we found only two pairs of TH-positive PPL2ab neurons were labelled by *UAS-mCD8::GFP*. Overexpression of TH in these two pairs of PPL2ab neurons enhanced the courtship index and courtship bout length in 40-day-old males (one-way ANOVA followed by Tukey's test, *P*<0.05) (Fig. 5d,e). These results identified the subset of PPL2ab neurons that are necessary and sufficient for DA-mediated courtship.

## Discussion

In addition to playing an important role in the regulation of sexuality^6^,^8^, DA also regulates other essential physiological functions in many organisms, including movement^1^, reward-related responses^3^, cognition^2^,^5^, attention^5^, circadian rhythms 4, motivation^2^,^3^, learning and memory^24^. Previous research has confirmed that DA contributes to many functions in flies^18^,^25^,^26^,^27^,^28^,^29^,^30^,^31^,^32^,^33^,^34^. In this study, we set out to determine the neural circuitry that underlies DA regulation of courtship through genetic and pharmacologic manipulations coupled with behavioural assays.

The courtship index is a quantitative expression of courtship duration measured as the percentage of time spent on courtship behaviour during each experimental period. Therefore, it quantifies the relative amount of courtship. Courtship bout length quantifies courtship episode duration, which indicates the degree to which the courtship period was fragmented between courtship and non-courtship behaviour. Therefore, it can be used as an indication of courtship quality. Our findings demonstrated, both quantitatively and qualitatively, an enhancement of male courtship display when DA levels were increased in PPL2ab neurons; we observed significant increases in both the courtship index (quantitative enhancement) and courtship bout length (qualitative enhancement). As courtship intensity does not always reflect mating propensity, we consistently observed no statistically significant differences in mating success rate when DA levels were increased in PPL2ab neurons in males (Supplementary Fig. 6). In contrast, when DA levels were downregulated in PPL2ab neurons, we observed a significant decrease in the courtship index and courtship bout length in males, indicated by a quantitative and qualitative inhibition of courtship display. Interestingly, DA release from a subset of PPL2ab neurons was sufficient to boost the male courtship sustainment in not only young, but also aged flies. Moreover, we demonstrated that DA-associated declines in PPL2ab neurons led to age-related diminutions in sexuality in males. This study therefore highlights the importance of DA levels in PPL2ab neurons, which have a potential role in regulating novel anti-senescence processes related to *Drosophila* male courtship activity.

Despite recent studies suggesting that neural DA-deficient flies show locomotion and sensory deficits^35^, altering DA levels in PPL2ab neurons did not affect motor activity and sensory inputs, including the senses of smell and taste, or even the lifespan (one-way ANOVA followed by Tukey's test, *P*>0.05) (Supplementary Fig. 7). This implies that PPL2ab neurons might regulate male sexuality specifically.

Our results thus may have revealed a specific sexual motivation circuit in which PPL2ab neurons regulate male sexual behaviour through unknown downstream neurons via DA signalling. The relationship of these neurons to sexually dimorphic neurons such as those expressing *fruitless* (*fru*) genes warrants further study, especially when considering that our data suggest there may be overlap in the projections of Fru-expressing neurons and the PPL2ab DA-expressing cells we identified. Fru-expressing neuronal circuitry have been tightly linked to *Drosophila* male courtship behaviour^36^, and Fru-expressing neurons are distributed in sensory, central and motor systems^37^,^38^,^39^,^40^,^41^,^42^. A male specific courtship inhibitory circuit begins in the Or67d sensory neurons that receive the external male-specific pheromone *cis*-vaccenyl acetate (cVA) from DA1 projection neurons. These cells then pass information to DC1 interneurons in the LH that then relay the cVA-induced signal to descending DN1 neurons. Finally, DN1 neurons send their axons to the thoracic ganglion, which causes male courtship inhibition^43^,^44^,^45^,^46^,^47^. It is worth noting that all the neurons in this circuitry are Fru-positive. In addition, the male-specific P1 cluster also contains Fru-positive neurons, which are potent activators of male courtship behaviour^48^. Arbours of P1 neurons may connect with P2b interneurons in the middle superior protocerebrum and extend descending fibres to the thoracic ganglia for courtship pattern generation^48^,^49^. In this study, we found that both of the PPL2ab subtypes identified in *murashka-1* flip-out clones similarly arborized their neurites to LH and one of the subtypes projected fibres to the medium superior protocerebrum, as well (Fig. 2g,h). On the basis of these observations, it is possible that PPL2ab neurons interact with the FruM circuitry. However, there are two other observations that suggest the PPL2ab circuitry may modulate courtship activity changes independently of FruM neurons. First, TH-positive PPL2ab neurons were not targeted by *fru-Gal4* (Supplementary Fig. 8a). Second, activating *fru-Gal4* neurons with *ReaChR* during the courtship assays did not affect courtship sustainment (one-way ANOVA followed by Tukey's test, *P*>0.05) (Supplementary Fig. 8b,c). Nevertheless, the Fru-expressing circuitry is broadly distributed in different sensory, central and motor systems. Further work is still needed to resolve this issue. Thus, thorough anatomical tracing of neural circuits originating from specific PPL2ab neurons may shed light on the pattern of sex-specific wiring of a neural circuitry in the fly brain. Nevertheless, our study provides novel insight into the roles of DA signalling in regulating male sexual behaviour over the lifespan, which advances efforts to understand the sexual brain.

## Methods

### Fly strains

Fly stocks were raised on standard cornmeal food and housed at 25 °C and 70% relative humidity on a 12:12 h light:dark cycle. The wild-type strain used was Canton-S w(CS10). The GAL4 and LexA driver strains used were *TH-Gal4* (ref. 17), *murashka-1-Gal4* (ref. 50), *c061-Gal4*;*MB-Gal80* (refs 27, 51), *NP2758-Gal4* (refs 34, 51), *NP1528-Gal4* (ref. 27), *MB-Gal80;krasavietz-Gal4* (ref. 51), *NP5272-Gal4* (ref. 27), *HL9-62-1N-Gal4* and *LG121-LexA* (a gift from Ann-Shyn Chiang, NTHU, Taiwan), *TH-C1-Gal4* (ref. 18), *TH-D1-Gal4* (ref. 18) (gift from Mark Wu, Johns Hopkins University), *NP3024-Gal4* and *NP5945-Gal4* (#113066, #105062; purchased from the *Drosophila* Genetic Resource Centre, Kyoto Institute of Technology, Japan), *UAS-thRNAi* (#108879; purchased from the Vienna *Drosophila* Resource Centre, Vienna, Austria). The *UAS*- and *LexAop*-effector strains used were *UAS–TH*^52^, *UAS-LexPR-attp40*^21^, *UAS*–*mCD8*::*GFP*, *UAS-ReaChR*, *LexAop-ReaChR* (#5137, #5130, #53748, #53747; purchased from the Bloomington *Drosophila* Stock Centre, University of Indiana, IN, USA). The *LexAop-mKO* and *fru-Gal4* (a gift from Ann-Shyn Chiang, NTHU, Taiwan) and the *LexAop-GeneSwitch-attp40* (ref. 21) were obtained from the laboratories that generated them. Our laboratory generated the *LexAop-TH-attp2*, *LexAop-TH-attp40*, *LexAop-thRNAi-attp2*, *LexAop-thRNAi-attp40*, *LexAop-FLP-attp3*, *UAS-frt-stop-frt-TH-attp40* transgenic lines used in this study. All brain images and behavioural analysis using transgenic expression utilized the progeny obtained from crossing Gal4 or LexA flies crossed to the indicated reporter or effector transgenes.

### Immunohistochemistry

Whole-mount immunolabelling of the adult brain was performed as previously described^21^. In brief, the dissected brains were fixed in 4% paraformaldehyde in 0.01 M phosphate-buffered saline (PBS) left on ice for 30 min before beginning the dissection. Next, the brains were transferred into penetration/blocking (P/B) buffer (2% Triton X-100, 0.02% NaN_3_, and 10% normal goat serum in PBS) at room temperature and kept under vacuum for 1 h. For immunohistochemical staining, the primary antibodies included 1:50 mouse 4F3 anti-Discs large antibody (anti-DLG) (Hybridoma Bank, University of Iowa) and 1:1,000 rat anti-tyrosine hydroxylase antibody (anti-TH, NB300-109, Novus Biologicals) at room temperature overnight. The secondary antibodies included biotin-conjugated goat anti-mouse IgGs (Invitrogen) and biotin-conjugated goat anti-rabbit IgGs (Invitrogen) that were diluted 1:200. Biotin-conjugated IgGs were probed by Alexa Fluor streptavidin 633 (1:1,000 dilution, Invitrogen). Immunostained brains were cleared and mounted in Focusclear (CelExplorer Labs, Taiwan) and imaged with a Zeiss LSM700 Confocal Microscope under a 40 × C-Apochromat water-immersion objective lens.

### Single-neuron imaging

For genetic FLP-out labelling, flies carrying the *hs-flp;+; muraskha-GAL4/UAS>rCD2,y*^*+*^*>mCD8*::*GFP* transgene were heat-shocked at 37 °C at the 4-day pupal stage for 10 min. Brain samples were incubated in a cocktail of primary antibodies including 1:500 rabbit anti-GFP (Molecular Probes) and 1:50 mouse 4F3 anti-DLG at room temperature overnight. After washing in PBS with 2% Triton X-100 (PBS-T) three times, the samples were incubated in 1:250 biotinylated goat anti-mouse or rabbit IgG (Molecular Probes) at room temperature for 1 day. Next, brain samples were washed and incubated in a cocktail solution including 1:1,000 goat anti-rabbit Alexa Fluor 488 and 1:1,000 Alexa Fluor 635 streptavidin (Molecular Probes) at room temperature overnight. After extensive washing in PBS, the brain samples were cleared and mounted in Focusclear (CelExplorer Labs, Taiwan) for imaging. The sample brains were imaged under a Zeiss LSM700 confocal microscope with a 40 × C-Apochromat water-immersion objective lens.

### Courtship assay

Naive males with no pretest social experience were collected on the day of eclosion and kept in individual test tubes in a 25 °C incubator with a 12 h L/D cycle. Target females were stored in groups (for example, 20 females per vial). Courtship assays were performed between hour 2 and hour 6 of the light cycle every day. A courtship chamber (1.2 cm diameter × 0.8 cm high) containing a layer yeast food was assembled in each cell. Flies were exposed to a mild level of CO_2_ for 10 min to immobilize them. Next, both test males and target females were transferred (3 days post-eclosion) to each cell of the chamber and video recorded (HDR-SR10 digital video camera, Sony, Japan) for 10 min. The courtship index was determined by the percentage of time before the copulation observation period that the tester male spent courting the target female (that is, tapping, following, wing vibration and attempted copulation)^53^,^54^. The average courtship bout length (duration) was calculated as the time spent by the male showing uninterrupted courtship behaviour.

### Statistical analysis

Statistical analyses were performed using SigmaPlot software version 12.0. All data were evaluated via one-way ANOVA followed by turkey's test or two-way ANOVA followed by a Bonferroni multiple-comparison test. Means within each data set followed by the same letters are not significantly different at the 5% level. All data were presented as mean+s.e.m.

### Additional methods

Details of transgene construction, the negative gravitaxis assay, proboscis extension response (PER) assay, the avoidance assay and quantitative measurements of DA by HPLC-ECD are provided in the Supplementary Online Methods.

## Additional information

**How to cite this article:** Kuo, S.-Y. *et al.* PPL2ab neurons restore sexual responses in aged **Drosophila** males through dopamine. *Nat. Commun.* 6:7490 doi: 10.1038/ncomms8490 (2015).

## Supplementary Material

Supplementary Information Supplementary Figures 1-8, Supplementary Table 1, Supplementary Methods and Supplementary References

Supplementary Movie 1 This movie shows examples of single PPL2ab neurons isolated from muraska-1 flip-out clones, as referred to in Figure 2g. Green corresponds to mCD8::GFP. Magenta represents immunostaining of the neuropil with an anti-DLG antibody.

Supplementary Movie 2 This movie shows examples of single PPL2ab neurons isolated from muraska-1 flip-out clones, as referred to in Figure 2h. Green corresponds to mCD8::GFP. Magenta represents immunostaining of the neuropil with an anti-DLG antibody.

Supplementary Data 1 NotI-frt-stop-frt-KpnI-TH-NotI DNA sequence.
