# Supplementary material for: PPL2ab neurons restore sexual responses in aged Drosophila males through dopamine
Source: Nat Commun. 2015 Jun 30;6:7490. doi: 10.1038/ncomms8490 (PMC4491191; doi:10.1038/ncomms8490)
Supplement: Supplementary Information — Supplementary Figures 1-8, Supplementary Table 1, Supplementary Methods and Supplementary References [file ncomms8490-s1.pdf]

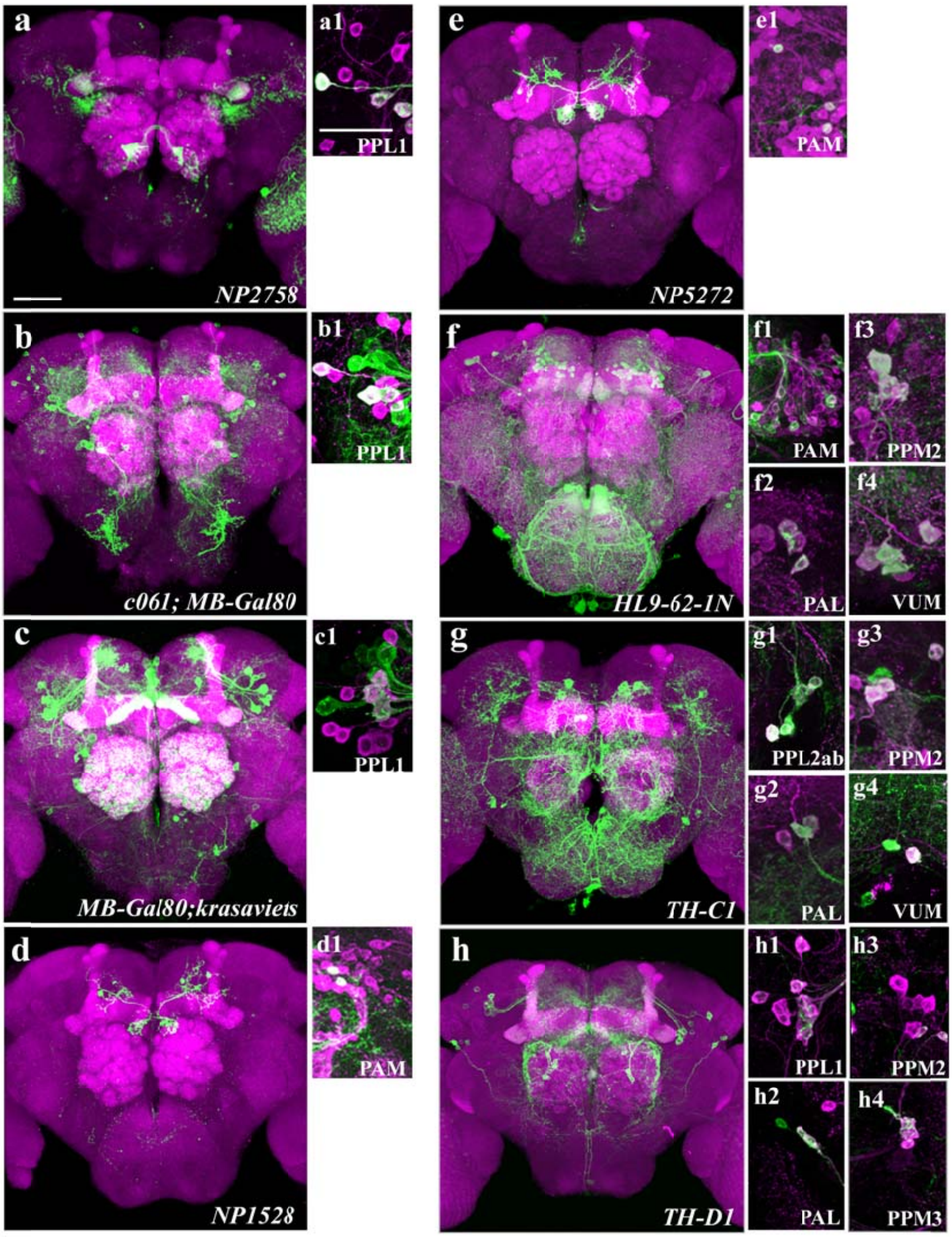

Supplemental Figure 1. Characterization of the Gal4 drivers used in this study.

Brain tissue of 10-day-old adult males from the various driver lines, (a) *NP2758-Gal4*; (b) *c061-Gal4;MB-Gal80*; (c) *MB-Gal80;krasavietz-Gal4*; (d) *NP1528-Gal4*; (e) *NP5272-Gal4*; (f) *HL9-62-1N-Gal4*; (g) *TH-C1-Gal4*; and (h) *TH-D1-Gal4,* reported by *UAS-mCD8::GFP* (green; a-h), were immunostained with an anti-DLG antibody to

label the neuropil (magenta; a-h) (frontal view, dorsal up) and the expression pattern of each driver was assessed. The scale bar is 50 µm. The cell bodies of PPL1 neurons were targeted (green in a1, b1, c1, and h1) by *NP1528-Gal4*, *NP5272-Gal4*, and *HL9-62-1N-Gal4*; PPL2ab neurons were targeted (green in g1) only by *TH-C1-Gal4*; PAL neurons were targeted (green in f2, g2 and h2) by *HL9-62-1N-Gal4*, *TH-C1-Gal4*, and *TH-D1-Gal4*; PPM2 neurons were targeted (green in f3, g3 and h3) by *HL9-62-1N-Gal4*, *TH-C1-Gal4*, and *TH-D1-Gal4*; VUM neurons were targeted (green in f4 and g4) by *HL9-62-1N-Gal4* and *TH-C1-Gal4*; and PPM3 neurons were targeted (green in h4) by only *TH-D1-Gal4*. Each neuron subset was confirmed to be DAergic by TH immunostaining (magenta in a1-e1, f1-f4, g1-4, and h1-4). Scale bar is 20 µm. Genotypes: (a) *NP2758-Gal4/y;UAS-mCD8::GFP/+;UAS-mCD8::GFP/+*; (b) *c061-Gal4/y;UAS-mCD8::GFP/MB-Gal80;+/UAS-mCD8::GFP*; (c) *+/y;UAS-mCD8::GFP/MB-Gal80;krasavietz-Gal4/UAS-mCD8::GFP*; (d) *+/y;UAS-mCD8::GFP/NP1528-Gal4;UAS-mCD8::GFP/+*; (e) *+/y;UAS-mCD8::GFP/NP5272-Gal4;UAS-mCD8::GFP/+*; (f) *+/y;UAS-mCD8::GFP/+; HL9-62-1N-Gal4/UAS-mCD8::GFP*; (g) *+/y;UAS-mCD8::GFP/TH-C1-Gal4;UAS-mCD8::GFP/+*; and (h) *+/y;UAS-mCD8::GFP/TH-D1-Gal4;UAS-mCD8::GFP/+*.

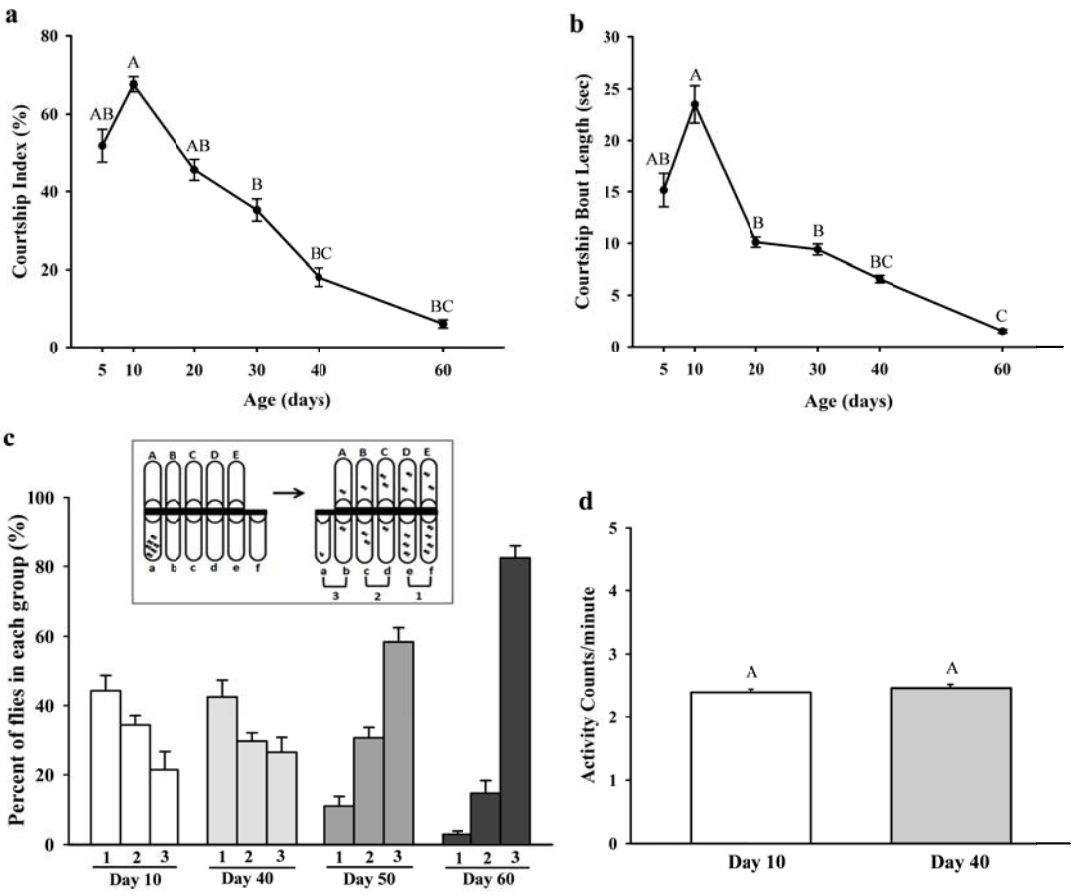

Supplemental Figure 2. Age-dependent changes in *Drosophila* male courtship sustainment. Wild-type (CS) male flies were collected shortly after eclosion, housed alone in vials for 5, 10, 20, 30, 40, and 60 days, and then courtship behavior towards 3-day old CS female flies was assayed for. Male courtship abilities observed at different ages are indicated by the courtship index (a) and courtship bout length (b), which are evaluations of male courtship sustainment. The changes in courtship index (a) and courtship bout length (b) levels peaked 10 days after eclosion in male flies and significant decreases were observed as age progressed through day 60. Each data point represents the mean + SEM of 16 tests. (c) We then tested motor activity using the negative gravitaxis assay to ensure that differences were not due to motor impairments. The inset box shows the layout to of the apparatus; a to f and A to E represent the lower and upper tubes, respectively. The brackets labeled 1-3 indicate the tubes that flies traveled to during testing, as shown in the bar graph (c).

Fifty-day-old (Day 50) and 60-day-old (Day 60) male flies showed significant impairment in negative gravitaxis compared to 40-day-old flies (Day 40). There was no significant difference in gravitaxis between 40-day-old (Day 40) and 10-day-old male flies. Values are presented as means + SEM (8 tests of 60 flies each). (d) The spontaneous motor activities in 10-day-old (10 Day; N = 124) and 40-day-old (40 Day; N = 66) male flies, were recorded by the *Drosophila* activity monitoring (DAM) system. Values are presented as + SEM. Means within each dataset followed by the same letters were not significantly different ($P < 0.05$) by a one-way ANOVA followed by a Tukey's test.

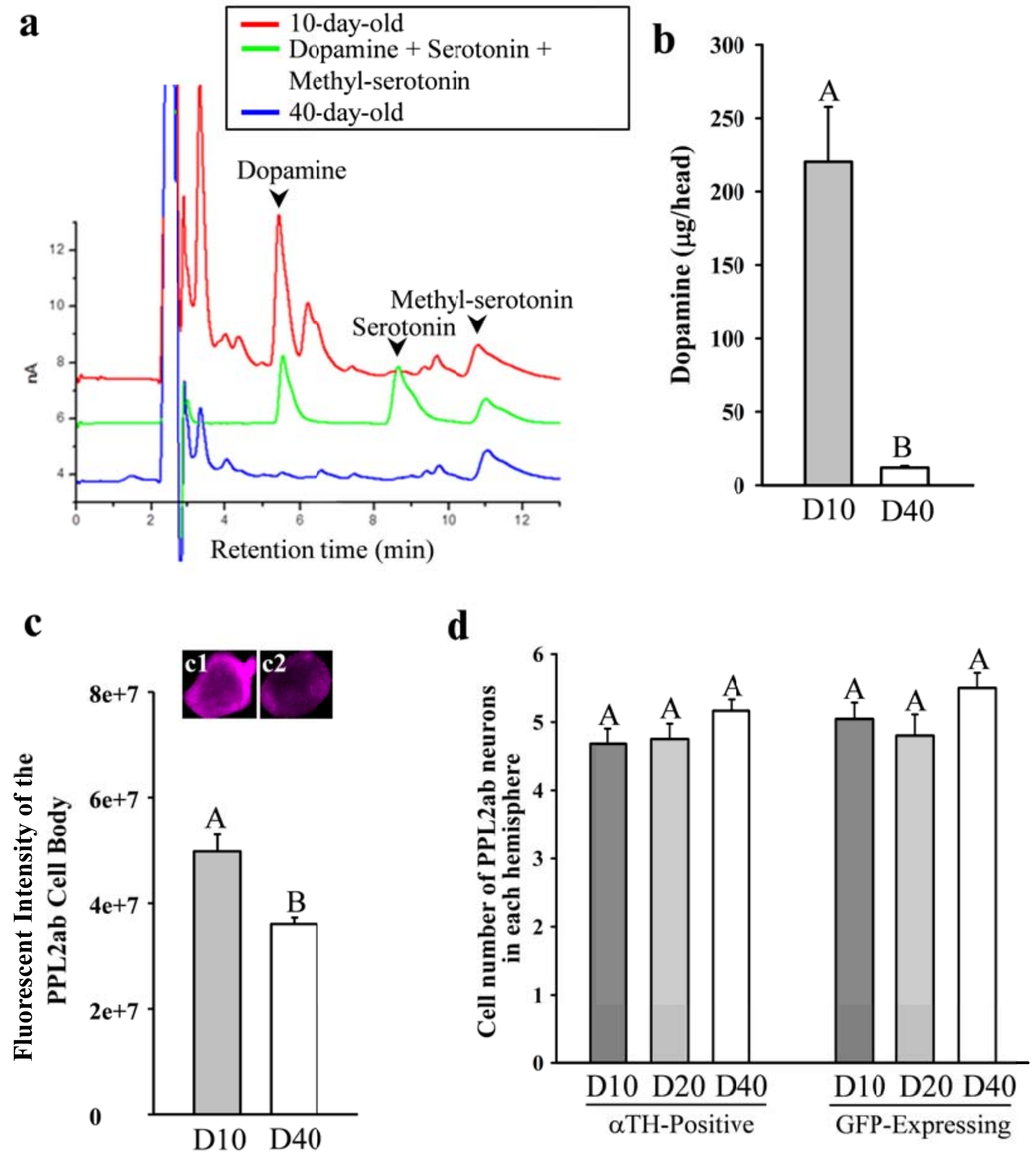

Supplemental Figure 3. Age-related changes in DA synthesis, without degeneration of DAergic PPL2ab neurons in aging flies. 10-day-old and 40-day-old flies (CS) were homogenized and individually measured for DA content by HPLC-ECD. (a) HPLC chromatograms of head extracts of young adult and aged flies are shown. The DA peaks are indicated by arrowheads. (b) DA levels (micrograms per head) in complete head extracts are shown. The DA levels observed in heads of 40-day-old fly (D10; gray bar) were significantly reduced compared with 40-day-old flies (D40; white bar). Each value represents mean + SEM (5 tests of 100

fly heads each). (c) Immunostaining for TH was reduced in PPL2ab neurons in 10-day-old males (c1) compared with 40-day-old males (c2). This was quantified and is shown in the bar graph for 10-day-old males (D10; gray bar) and 40-day-old males (D40; white bar), as well. (d) Mean number of DA neurons per hemisphere in protocerebral PPL2ab clusters. We counted DA neuron numbers/cluster in heterozygous male *TH-Gal4* > *UAS-mCD8*::*GFP* brains at day 10 (D10), day 20 (D20), and day 40 (D40) (n = 22 hemispheres each). The neuron numbers were quantified based on either anti-TH immunostaining [plot on left in (d)], or GFP labeling [plot on right in (d)]. Values are presented as mean + SEM. Means within each dataset followed by the same letters were not significantly different at $P < 0.05$ by a one-way ANOVA followed by a Tukey's test.

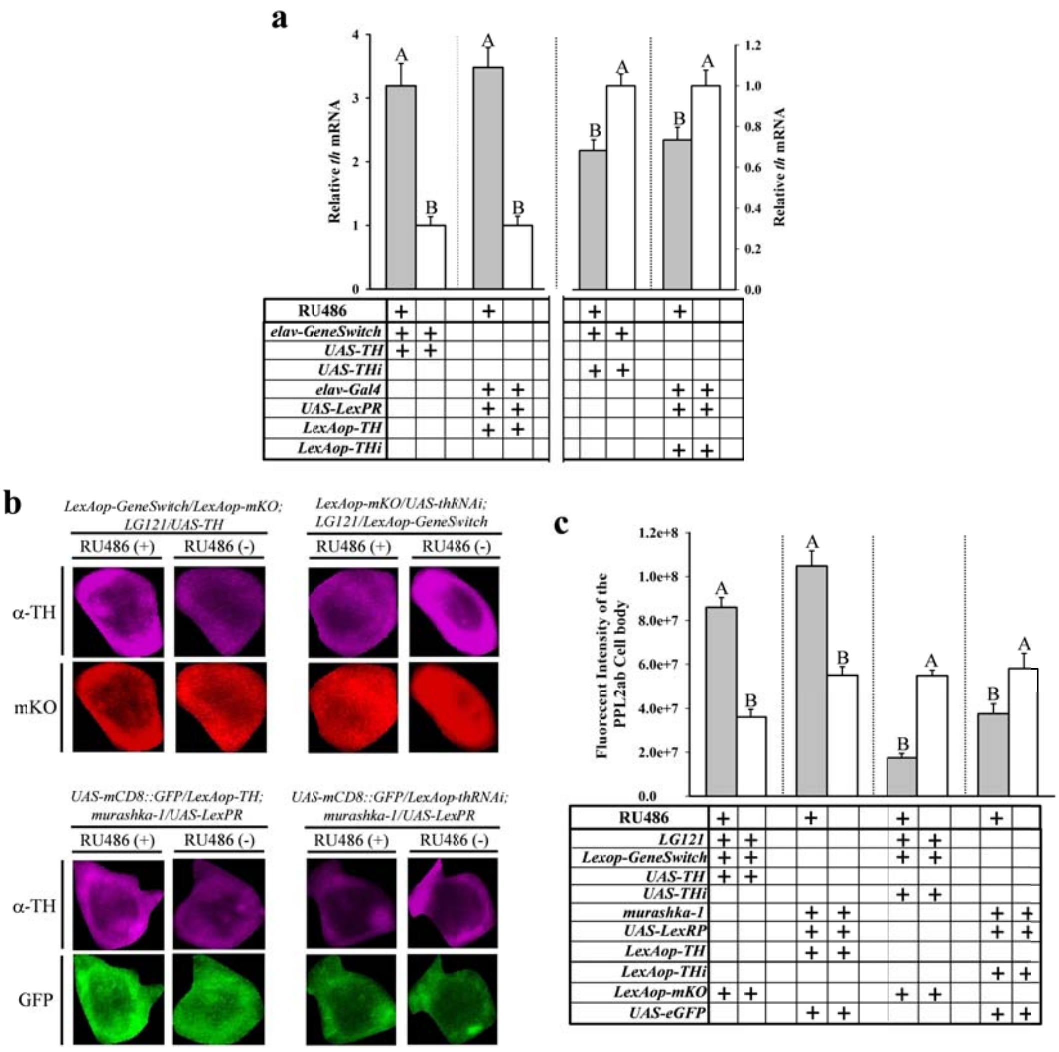

Supplemental Figure 4. Characterization of the effectiveness of knockdown or overexpression in the *th*RNAi and *th* effector lines, respectively. (a) RT-qPCR showing altered amount of *th* transcripts in the fly head after the induction of *th* overexpression or *th* knockdown in 5-day-old males fed 1.5 mM RU486 (+) for 5 days. Relative mRNA expression was determined using *rp49*. The *th* transcripts and DA level were significantly increased in the flies that carried *+/y;+/+;elav-GeneSwitch/UAS-TH* or *elav-Gal4/y; LexAop-TH/+;UAS-LexPR/+* alleles. In contrast, the *th* transcripts and DA levels were significantly reduced in the flies that carried *+/y; UAS-thRNAi/+;elav-GeneSwitch/UAS-Dcr2* or *elav-Gal4/y;LexAop-thRNAi/+;UAS-LexPR/UAS-Drc2* compared with flies of the

same genotype that did not receive RU486 treatment. Each column represents the mean of 5 tests. Forward primer (5’-TCGAGAAGCTGTCCACGGTATA) and reverse primer (5’- CTGACCATGTTCCTTGCAGAGA), respectively, used for *th* amplification. (b) After feeding male flies 1.5 mM RU486 (+) beginning at the age of 5 days or 35 days, TH immunolabeling similarly indicated an increased amount of TH protein in PPL2ab neurons in flies carrying *+/y;LexAop-GeneSwitch/LexAop-mKO;LG121/UAS-TH* or *+/y; LexAop-TH/UAS-mCD8::GFP;murashka-1-Gal4/UAS-LexPR*. (c) These differences in TH fluorescent immunostaining levels between experimental and control groups were quantified. The results shown in each bar represent the mean of 10 tests. Values are presented as mean + SEM. Pairs of bars with the same letters were not significantly different, whereas pairs of bars with different letters (A and B) were significantly different, as defined by $P < 0.05$ determined by a one-way ANOVA followed by a Tukey’s test per grouped columns (separated by a dashed line).

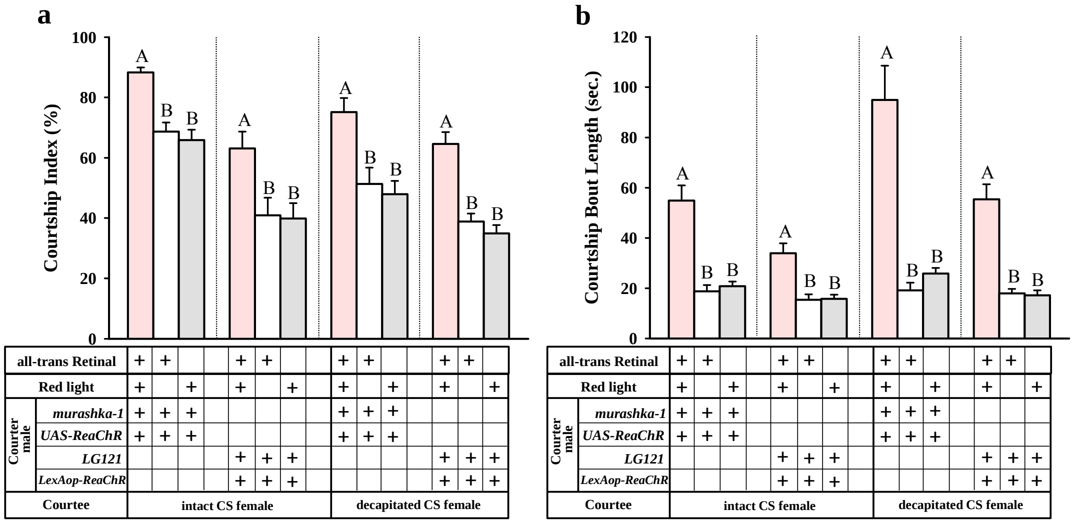

Supplemental Figure 5. Activating PPL2ab neurons improves male courtship sustainment. The courtship index and courtship bout length of 10-day-old male flies toward 3-day-old intact CS or decapitated CS females was determined during optogenetic activation of TH neuron subtypes. There were significant differences in the courtship index (a) and courtship bout length (b) observed in flies that carried *murashka-1-Gal4>UAS-ReaChR* and *LG121-LexA>LexAop-ReaChR* compared with flies of the same genotype that were either not exposed to red light or did not receive all-trans-retinal treatment. Each column represents the mean of 18 tests. Pairs of bars with the same letters were not significantly different, whereas pairs of bars with different letters (A and B) were significantly different as defined by $P < 0.05$ determined by a one-way ANOVA followed by a Tukey's test per grouped columns (separated by a dashed line). Genotypes: (1) *+/+;UAS-ReaChR/+;murashka-1-Gal4/+*; and (2) *+/+;LexAop-ReaChR/+;LG121-LexA/+*.

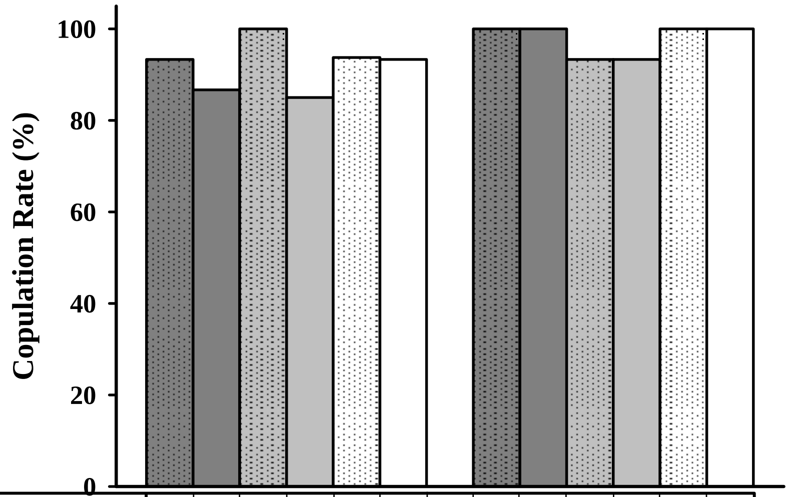

RU486		+		+		+			+		+		+	
Courter male	*murashka-1*	+	+	+	+									
	UAS-LexPR	+	+	+	+									
	LexAop-TH	+	+			+	+							
	LG121								+	+	+	+		
	LexAop-GS								+	+	+	+		
	UAS-TH								+	+			+	+
Courtee		intact CS female												

Supplemental Figure 6. Increasing DA levels in PPL2ab neurons does not affect copulation rate. The LexPR/*LexAop* (or GeneSwitch/*UAS*) inducible system, which was applied to temporally increase DA levels specifically in *murashka-1-Gal4-* and *LG121-LexA*-expressing neurons. Males aged 5 days were fed 1.5 mM RU486 (+) for 5 days, and the mating success rate towards 5-day-old CS females was counted at the 10 day-old time point toward 15 pair tests during the 10 min experimental period. There were no significant differences in the mating success rate in the flies that received RU486 and carried the *murashka-1-Gal4>UAS-LexPR; LexAop-TH* or *LG121-LexA>LexAop-GeneSwitch;UAS-TH* compared with flies of the same genotype that did not receive RU486 treatment, as well as for the corresponding heterozygous driver and effector genotypes.

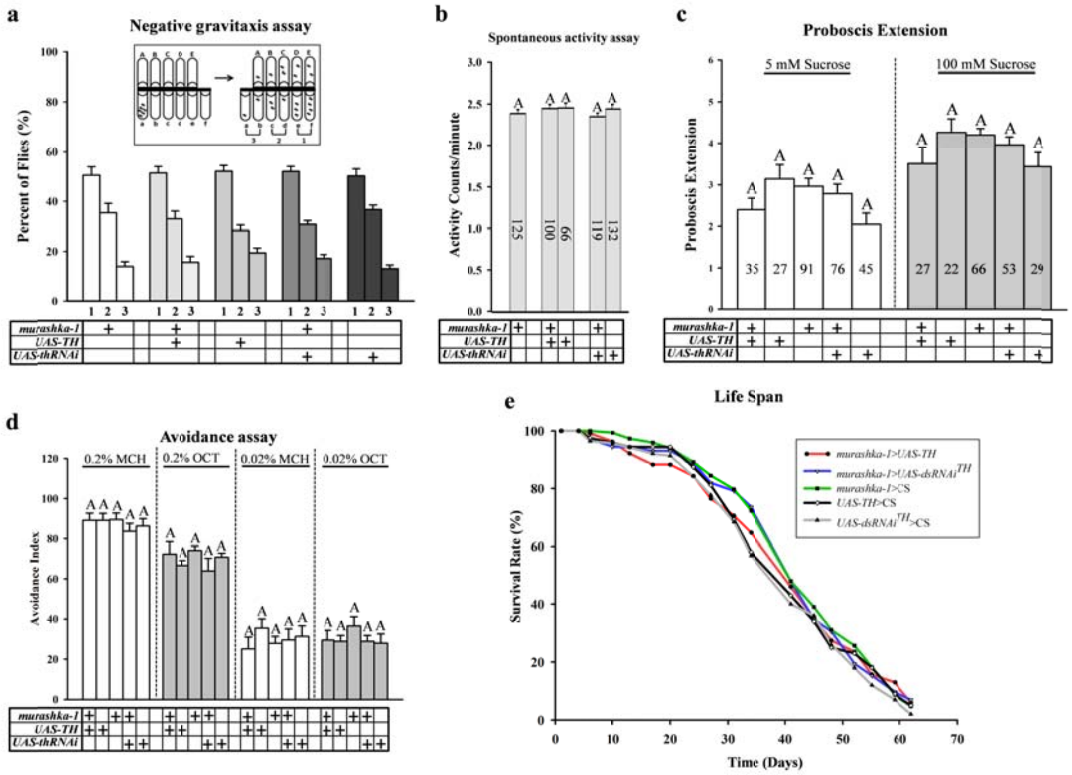

Supplemental Figure 7. Genetic manipulation of TH level in PPL2ab neurons does not affect nonsexual behaviors. (a) Genetic manipulations of DA in a PPL2ab cluster did not affect negative gravitaxis (climbing activity). Ten-day-old male flies that carried *murashka-1-Gal4>UAS–TH* or *murashka-1-Gal4>UAS–thRNAi* exhibited normal climbing activity compared with control flies. The values are presented as mean + SEM (*n* = 12 tests). (b) Genetic manipulations of DA in a PPL2ab cluster did not affect spontaneous motor activity recorded by DAMS. Ten-day-old male flies that carried *murashka-1-Gal4>UAS–TH* or *murashka-1-Gal4>UAS–thRNAi* exhibited normal spontaneous motor activity compared with control flies. The values are means ± SEM (the *n* for each group is shown in each bar). (c) Different concentrations of sucrose were used to test gustatory detection abilities in 10 day-old *murashka-1-Gal4>UAS–TH* flies, *murashka-1>UAS–thRNAi* flies, and control flies, revealing no significant differences. Values are presented as means ± SEM (the *n* for each group is shown in each bar). (d) Different concentrations of

4-methylcyclohexanol (MCH) and 3-octanol (OCT) were used to measure the olfactory detection ability in 10 day-old *murashka-1-Gal4>UAS–TH, murashka-1-Gal4>UAS–thRNAi*, and control flies, revealing no significant differences. The values are presented as means + SEM ($n = 6$ tests for 0.2% odorants and $n = 8$ for 0.02% odorants). (e) Survival rate analysis for each genotype. Flies were fed standard cornmeal food at 25 °C with enumeration and transfer of survivors to fresh bottles every 2–3 days. The percentage of surviving flies was calculated and plotted. The mean values within each dataset followed by the same letters were not significantly different per each column group at a threshold of $P < 0.05$ determined by a one-way ANOVA followed by a Tukey's test per grouped columns (separated by a dashed line). Genotypes: (1) *+/+;UAS-TH/+;murashka-1-Gal4/+*; (2) *+/+;UAS-TH/+;+/+*; (3) *+/+;+/+;murashka-1-Gal4/+*; (4) *+/+;UAS-thRNAi/+;murashka-1-Gal4/+*; and (5) *+/+;UAS-thRNAi/+;+/+*.

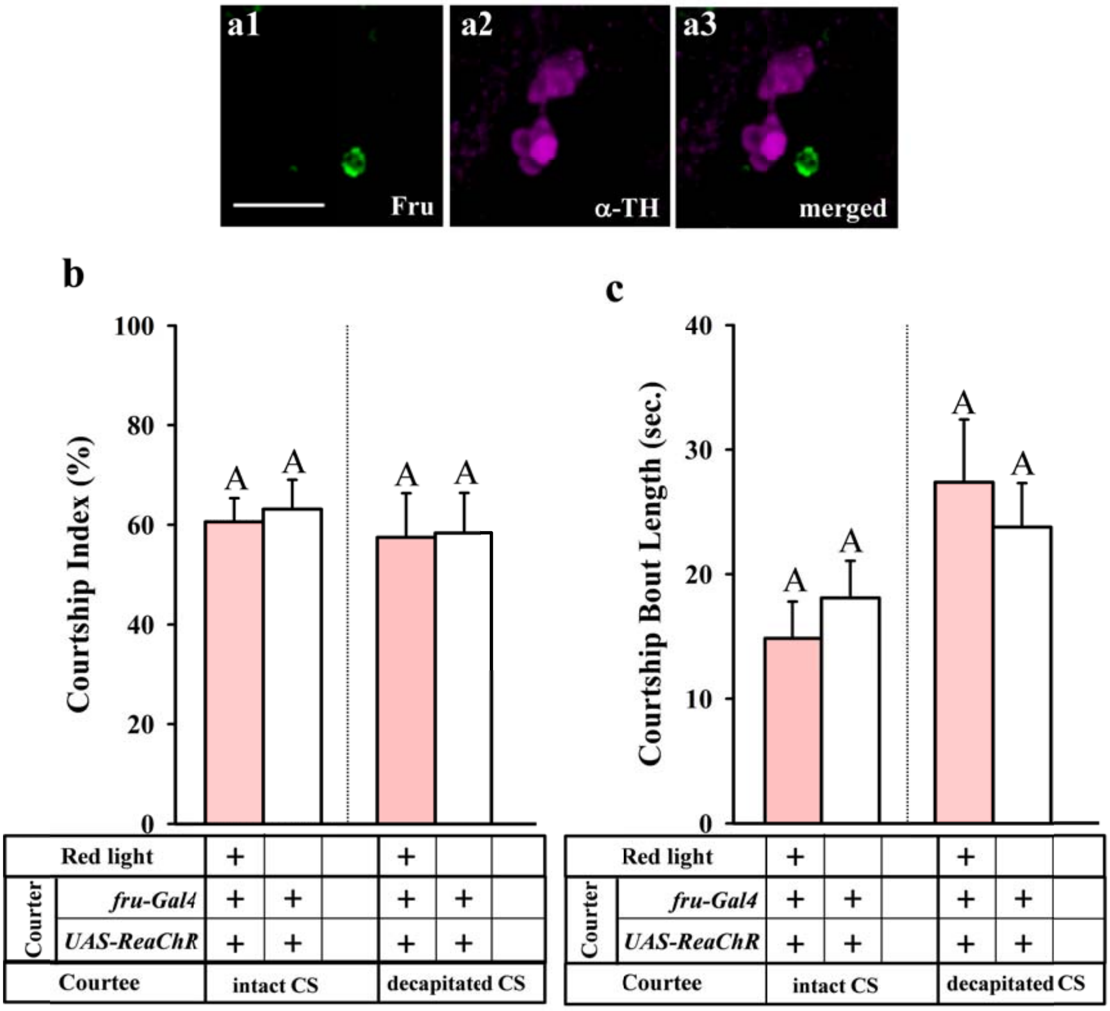

Supplemental Figure 8. Activating *fru-Gal4* expression circuitry failed to improve courtship sustainment. (a) The cell bodies of TH-positive PPL2ab neurons in *fru-Gal4* (green in a1) did not express TH, as indicated by immunostaining (magenta in a2) and a merged image in a3 of a representative 10-day-old *fru-Gal4/y;UAS-mCD8::GFP/+;UAS-mCD8::GFP* fly. The scale bars are 20 μm. (b, c) The courtship index and courtship bout length toward 3-day-old intact CS females or decapitated CS females were analyzed in 10-day-old male flies. There were no significant differences in the courtship index and courtship bout length in the flies that carried *fru-Gal4>UAS-ReaChR* compared with flies of the same genotype that were not subjected to red light. Each bar represents the mean of 18 tests. The error bars indicate + SEM. Pairs of bars with the same letters were not significantly different,

whereas pairs of bars with different letters were significantly different as defined by $P < 0.05$ determined by a one-way ANOVA followed by a Tukey's test per grouped columns (separated by a dashed line). Genotypes: *fru-Gal4/y;UAS-ReaChR/+;+/+*.

Table 1. Quantification of TH-positive neurons labeled by each driver

Driver	DA neurons (mean ± SEM)								
	per cerebral hemisphere								per cerebra
	PAM	PAL	PPL1	PPL2ab	PPL2c	PPM1	PPM2	PPM3	VUM
TH-C1	**11 ± 0.86**	**3 ± 0.19**	**0**	**6.13 ± 0.55**	**0**	**1.13 ± 0.13**	**4.38 ± 0.26**	**0**	**2.4 ± 0.34**
TH-D1	**0**	2	**7.13 ± 0.44**	**0**	**1.75 ± 0.25**	**0**	**1.63 ± 0.18**	**4.63 ± 0.38**	**0**
murashka-1	**0**	**0**	**0**	**2.75 ± 0.25**	**0**	**0**	**2.5 ± 0.5**	**0**	**0**
LG121	**0**	**0**	**0**	**5.3 ± 0.21**	**0**	**0**	**0**	**0**	**0**
NP3024	**0**	**0**	**0**	**2.5 ± 0.34**	**0**	**0**	**2.13 ± 0.23**	**1.63 ± 0.38**	**0**
NP5945	**0**	**0**	**0**	**2.75 ± 0.48**	**0**	**0**	**3.25 ± 0.48**	**0**	**0**
c061;MB-Gal80	**0**	**0**	11	**0**		**0**		1	**0**
NP2758	**0**	**0**	**2.67 ± 0.17**	**0**	**0**	**0**	**3.5 ± 0.31**	**0**	**0**
MB-Gal80; krasaviets	**0**	**0**	9	**0**	**0**	**0**	**0**	**0**	**0**
HL9-62-1N	**28.4 ± 0.82**	**3 ± 0.26**	**0**	**0**	**1.7 ± 0.26**	**0**	**7.7 ± 0.50**	**0.9 ± 0.18**	**4.3 ± 0.33**
NP1528	**3.25 ± 0.23**	**0**	**0**	**0**	**0**	**0**	**0**	**0**	**0**
NP5272	**1.75 ± 0.25**	**0**	**0**	**0**	**0**	**0**	**0**	**0**	**0**

TH-positive cells targeted by each driver, as determined by analysis of coexpression of eGFP with immunostaining for TH. The table reports the average numbers of neurons ± SEM counted per driver line.

Supplementary Methods

Transgene Construction. Cloning of pLexAop-AI-TH: The pUAST-TH clone was provided from Sean B. Carroll (HHMI and University of Wisconsin). This clone contains *Eco*RI and *Kpn*I cutting sites for release of the full TH gene; this region was cloned into the *Eco*RI and *Kpn*I sites of a pLexAop-AI vector[1] to create the pLexAop-AI-TH transgene. Cloning of pLexAop-thRNAi: The pLexAop-AII vector was designed to express dsRNAi under control by the LexA/*LexAop* system. This plasmid includes the attB site, gypsy insulator, and white intron 2, and it is derived from the pLexAop-AI plasmid. The pWIZ[2] clone was purchased from the *Drosophila* Genomics Resource Center (#1008). This clone contains *Eco*RI and *Xba*I cutting sites for release of the *white* intron and its 3' and 5' flanked restriction sites; this region was cloned into the *Eco*RI and *Xba*I sites of pLexAop-AI to create the pLexAop-AII vector. The *th* partial fragment was repeatedly cloned into the *Xba*I and *Eco*RI sites of the pLexAop-AII vector to create the pLexAop-AII-thRNAi transgene. The *th* cDNA clone (pLexAop-AI-TH) using a previously described PCR protocol. Two unique primers (*Xba*I: 5'-TCTAGAGTTGGACATGACCCGTGGCAAT-3' and *Xba*I: 5'-TCTAGAGTCCCGGGCAGTCAAAAGACCG-3') were used to generate a PCR product corresponding to bases 405-918 of the *th* coding sequence. The amplified PCR fragment was sub-cloned into pGEM-T-easy (Promega, UAS) to create the pGEM-T-easy-$TH_{405\text{-}918}$ clone for sequencing and further sub-cloning. The "sense" fragment was released from pGEM-T-easy-$TH_{405\text{-}918}$ by *Xba*I digestion and cloned into the *Xba*I sites of the pLexAop-AII vector to create the intermediate pLexAop-AII-$TH_{405\text{-}918}$ clone. Finally, the inverted cDNA sequence ("antisense") was released from pGEM-T-easy-$TH_{405\text{-}918}$ by *Eco*RI digestion and then cloned into the *Eco*RI sites of the pLexAop-AII-$TH_{405\text{-}918}$ to create the pLexAop-thRNAi transgene. Cloning of p10XUAS-frt-stop-frt-TH: We utilized an efficient long gene synthesis

method to synthesize the frt-stop-frt-TH DNA fragment (Supplementary Data 1) (commissioned in Genomics BioSci & Tech, Taiwan). On both sides, we designed a *Not*I cutting site in synthesized DNA fragments for cloning into the *Not*I sites of pJFRC81-10XUAS-IVS-Syn21-GFP-p10 (Addgene, plasmid #36432) to create the p10XUAS-frt-stop-frt-TH transgene. We inserted these transgenes with phiC31 recombinase into specific *attP2, attp3*, or *attP40* sites[3] of Canton-S w(CS10) flies using standard methods.

Behavior. *Negative gravitaxis assay (climbing assay)*: Gravitactic behavior was evaluated as previously described[4]. To determine the activity trends of the flies, we summed the number of the flies in tubes a and b, tubes c and d, and tubes e and f, as shown in Supplementary Fig. 3a. These data are represented in a bar graph (see Supplementary Fig. 3a).

Spontaneous activity assay: We measured the spontaneous waking activity for each genotype by recording the locomotion of males for 24 continuous hours using the *Drosophila* Activity Monitoring (DAM) system (Trikinetics, Waltham, MA)[5, 6]. Each fly was housed separately in an activity monitor tube. The DAM system uses an infrared beam to detect movement in the monitor tube. The movement is recorded as activity counts per 1-min interval. A sleep program was used to compute waking activity in counts per waking minute. Sleep was defined as inactivity that lasted 5 min or longer.

Proboscis extension response (PER) assay: Proboscis extension was assayed in 40-day-old males as previously described[7] with the following modifications. Flies aged 39 days were starved for 24 hours, immobilized by chilling on ice, and mounted ventral-side-up using non-toxic double adhesive tape. Flies were tested by touching a drop of sucrose solution on a pipette tip to the tarsi of the foreleg and scored for full proboscis extension. Each fly was given five trials of same substance in each sucrose

concentration (5 mM and 100 mM sucrose) with water application in-between trials. The average number of extensions was calculated for each genotype at each concentration by tallying the number of flies that responded to the test. *Odor Avoidance assay*: Odor-avoidance responses to 3-octanol (OCT) or to 4-methycyclohexanol (MCH) at two different concentrations (0.2% and 0.02%) were quantified individually. Approximately 100 flies were given a choice between either OCT or MCH versus "fresh" room air in the T-maze. After 2 min, the number of flies in each arm of the T-maze was counted, and an avoidance index value was calculated using the number of flies that chose air versus OCT or air versus MCH: (air − OCT)/(air + OCT)×100 or (air − MCH)/(air + MCH)×100 [8, 9]. *Lifespan assay*: The fly lifespan assay was carried out according to the method by Wang et al[10].

Quantitative Measurements of DA. We used an Agilent 1200 Series of high-performance liquid chromatography coupled with an electrochemical detector system (HPLC-ECD). Sample preparation was similar to that reported previously[11, 12]. Briefly, 100 heads of 10- and 40-day-old adult flies were homogenized in 100 µl ice-cold solution containing 50 mM citrate/acetate (pH 4.5), 11 mM 1-decanesulfonic acid (DSA) and 20% acetonitrile. After an equal volume of ice-cold methanol was added, the solution was run through a 0.22-µm Spin-X filter (Costar). Next, 20 µl of the homogenate (equivalent to 10 heads) and an internal standard of 5-hydroxy-N-methyltryptamine (800 pg/ml) were injected. Reverse phase chromatography was performed using a ZORBAX Rx-C18 column (250 mm × 4.6 mm i.d.) with a 5-µm particle size and 80-Å pore size (Agilent). A reliance cartridge guard column (12.5 mm × 4.6 mm i.d.) (Agilent) was placed between the injector and the analytical column. The mobile phase was delivered at a flow rate of 1 ml/min to separate and detect DA. The level of DA was quantified based on a standard curve using HPCORE Chem-Station software (Agilent).

Supplementary References

1. Kuo, S.Y., *et al.* A hormone receptor-based transactivator bridges different binary systems to precisely control spatial-temporal gene expression in Drosophila. *PLoS One* **7**, e50855 (2012).
2. Lee, Y.S., Carthew R.W. Making a better RNAi vector for Drosophila: use of intron spacers. *Methods* **30**, 322-329 (2003).
3. Markstein, M., Pitsouli C., Villalta C., Celniker S.E., Perrimon N. Exploiting position effects and the gypsy retrovirus insulator to engineer precisely expressed transgenes. *Nat. Genet.* **40**, 476-483 (2008).
4. Inagaki, H.K., Kamikouchi A., Ito K. Methods for quantifying simple gravity sensing in Drosophila melanogaster. *Nature protocols* **5**, 20-25 (2010).
5. Hendricks, J.C., *et al.* Rest in Drosophila is a sleep-like state. *Neuron* **25**, 129-138 (2000).
6. Shaw, P.J., Cirelli, C., Greenspan, R.J., Tononi, G. Correlates of sleep and waking in Drosophila melanogaster. *Science* **287**, 1834-1837 (2000).
7. Heimbeck, G., Bugnon, V., Gendre, N., Keller, A., Stocker, R.F. A central neural circuit for experience-independent olfactory and courtship behavior in Drosophila melanogaster. *Proc. Natl. Acad. Sci. U.S.A.* **98**, 15336-15341 (2001).
8. Tully., T., Quinn W.G. Classical conditioning and retention in normal and mutant Drosophila melanogaster. *J. Comp. Physiol. A* **157**, 263-277 (1985).
9. Ferris J., Ge H., Liu, L., Roman G. G(o) signaling is required for Drosophila associative learning. *Nat. Neurosci.* **9**, 1036-1040 (2006).
10. Wang, C.T., *et al.* Reduced neuronal expression of ribose-5-phosphate isomerase enhances tolerance to oxidative stress, extends lifespan, and attenuates polyglutamine toxicity in Drosophila. *Aging Cell* **11**, 93-103 (2012).
11. Hardie, S.L., Hirsh, J. An improved method for the separation and detection of biogenic amines in adult Drosophila brain extracts by high performance liquid chromatography. *J. Neurosci. Methods.* **153**, 243-249 (2006).
12. Lee P.T., *et al.* Serotonin-mushroom body circuit modulating the formation of anesthesia-resistant memory in Drosophila. *Proc. Natl. Acad. Sci. U.S.A.* **108**, 13794-13799 (2011).
