# Supplementary material for: PPL2ab neurons restore sexual responses in aged Drosophila males through dopamine
Source: Nat Commun. 2015 Jun 30;6:7490. doi: 10.1038/ncomms8490 (PMC4491191; doi:10.1038/ncomms8490)
Supplement: Supplementary Data 1 — NotI-frt-stop-frt-KpnI-TH-NotI DNA sequence. [file ncomms8490-s4.docx]

**Supplementary Data**

**1. *NotI-frt-stop-frt-KpnI-TH-NotI* DNA sequence**

GCGGCCGCGAAGTTCCTATACTTTCTAGAGAATAGGAACTTCTAACGTAAGCTAGCTAGACCGGTGTCGACTAAAGCCAAATAGAAAATTATTCAGTTCCTGGCTTAAGTTTTTAAAAGTGATATTATTTATTTGGTTGTAACCAACCAAAAGAATGTAAATAACTAATACATAATTATGTTAGTTTTAAGTTAGCAACAAATTGATTTTAGCTATATTAGCTACTTGGTTAATAAATAGAATATATTTATTTAAAGATAATTGCGTTTTTATTGTCAGGGAGTGAGTTTGCTTAAAAACTCGTTTAGGTTTGTCCTCCCGAAATTATTTATTTAAATGCGATGGAGAGTTGGCGCCGAATCGAAAACTTTACGCGCTTAAAAGCACGAGTTGGCATCCCTAACGCGTAGGATCTTTGTGAAGGAACCTTACTTCTGTGGTGTGACATAATTGGACAAACTACCTACAGAGATTTAAAGCTCTAAGGTAAATATAAAATTTTTAAGTGTATAATGTGTTAAACTACTGATTCTAATTGTTTGTGTATTTTAGATTCCAACCTATGGAACTGATGAATGGGAGCAGTGGTGGAATGCCTTTAATGAGGAAAACCTGTTTTGCTCAGAAGAAATGCCATCTAGTGATGATGAGGCTACTGCTGACTCTCAACATTCTACTCCTCCAAAAAAGAAGAGAAAGGTAGAAGACCCCAAGGACTTTCCTTCAGAATTGCTAAGTTTTTTGAGTCATGCTGTGTTTAGTAATAGAACTCTTGCTTGCTTTGCTATTTACACCACAAAGGAAAAAGCTGCACTGCTATACAAGAAAATTATGGAAAAATATTTGATGTATAGTGCCTTGACTAGAGATCATAATCAGCCATACCACATTTGTAGAGGTTTTACTTGCTTTAAAAAACCTCCCACACCTCCCCCTGAACCTGAAACATAAAATGAATGCAATTGTTGTTGTTAACTTGTTTATTGCAGCTTATAATGGTTACAAATAAAGCAATAGCATCACAAATTTCACAAATAAAGCATTTTTTTCACTGCATTCTAGTTGTGGTTTGTCCAAACTCATCAATGTATCTTATCATGTCTGGATCACTAGTGATCTGGCCGGGAAGTTCCTATACTTTCTAGAGAATAGGAACTTCGGTACCAATCAAAATGATGGCCGTTGCAGCAGCCCAAAAGAACCGCGAGATGTTCGCCATCAAGAAATCCTACAGTATTGAGAATGGCTATCCATCCCGCCGTCGCAGCCTGGTGGATGATGCCCGTTTCGAGACCCTGGTGGTCAAGCAGACCAAACAAACCGTCCTCGAGGAGGCCCGCAGCAAGGCAAATGATTACGGTCTCACCGAGGACGAGATTTTGTTGGCCAATGCCGCCTCCGAATCCTCGGATGCCGAGGCTGCCATGCAGAGTGCCGCTTTGGTGGTCCGCCTCAAGGAGGGCATCTCCTCCTTGGGTCGCATCCTCAAGGCCATCGAAACCTTCCACGGCACCGTCCAGCATGTGGAGTCCCGTCAGTCGCGCGTGGAGGGCGTGGACCACGATGTCCTCATCAAGTTGGACATGACCCGTGGCAATCTGCTGCAGCTGATCCGCTCCCTCAGGCAGTCGGGCTCCTTCAGCAGCATGAATCTGATGGCCGACAATAACTTGAATGTCAAGGCTCCGTGGTTCCCCAAGCACGCCTCCGAATTGGATAACTGCAACCATCTGATGACCAAGTACGAGCCCGATTTGGACATGAACCACCCCGGATTCGCCGACAAGGTATACCGCCAGCGTCGCAAGGAAATTGCCGAGATCGCATTCGCCTACAAGTACGGAGACCCGATCCCATTCATCGACTACTCCGATGTGGAGGTCAAGACCTGGCGCTCGGTGTTCAAGACCGTTCAGGATCTGGCTCCCAAGCACGCCTGTGCCGAGTACCGGGCCGCCTTCCAGAAGCTCCAGGATGAGCAGATCTTCGTGGAGACCCGTCTGCCCCAGTTGCAGGAGATGTCCGACTTTCTGCGCAAGAACACCGGATTCTCTCTCCGTCCTGCCGCCGGTCTTTTGACTGCCCGGGACTTCCTTGCCTCCTTGGCCTTCCGCATCTTCCAGAGCACCCAGTATGTGCGCCACGTTAACTCACCATACCACACCCCCGAGCCCGACTCCATTCACGAGCTGCTGGGTCACATGCCCCTGCTGGCCGATCCCAGCTTCGCCCAGTTCTCGCAGGAGATTGGACTGGCCTCGCTGGGTGCCTCCGACGAAGAAATCGAGAAGCTGTCCACGGTATACTGGTTCACTGTTGAGTTCGGTCTCTGCAAGGAACATGGTCAGATCAAGGCCTACGGTGCTGGACTCCTGAGCTCCTACGGTGAGCTGCTCCATGCCATCAGCGACAAGTGCGAGCACCGCGCCTTCGAGCCCGCATCCACCGCCGTGCAGCCCTACCAGGATCAGGAGTACCAGCCCATCTACTATGTGGCCGAGAGCTTCGAGGATGCCAAGGACAAGTTCCGTCGCTGGGTGAGCACCATGTCGCGTCCATTCGAGGTGCGTTTCAACCCGCACACCGAGCGCGTCGAGGTGCTGGACTCCGTCGACAAGCTGGAGACTCTGGTGCACCAGATGAACACGGAGATTTTGCATCTGACCAACGCCATCTCCAAGTTGCGACGCCCGTTCTAAGCGGCCGC
